# Optimizing the Use of iPSC-CMs for Cardiac Regeneration in Animal Models

**DOI:** 10.3390/ani10091561

**Published:** 2020-09-02

**Authors:** Alexandra Bizy, Matthew Klos

**Affiliations:** 1 Department of Biomedical Sciences, Faculty of Health Sciences, Universidad CEU Cardenal Herrera, CEU Universities, Moncada, 46113 Valencia, Spain; alexandra.bizy@uchceu.es; 2 Pediatric Cardiac and Thoracic Surgery, University Hospitals Cleveland Medical Center, Cleveland, OH 44106, USA

**Keywords:** heart failure, cardiac regeneration, iPSC-CMs, metabolism

## Abstract

**Simple Summary:**

In 2006, the first induced pluripotent stem cells were generated by reprogramming skin cells. Induced pluripotent stem cells undergo fast cell division, can differentiate into many different cell types, can be patient-specific, and do not raise ethical issues. Thus, they offer great promise as in vitro disease models, drug toxicity testing platforms, and for autologous tissue regeneration. Heart failure is one of the major causes of death worldwide. It occurs when the heart cannot meet the body’s metabolic demands. Induced pluripotent stem cells can be differentiated into cardiac myocytes, can form patches resembling native cardiac tissue, and can engraft to the damaged heart. However, despite correct host/graft coupling, most animal studies demonstrate an arrhythmogenicity of the engrafted tissue and variable survival. This is partially because of the heterogeneity and immaturity of the cells. New evidence suggests that by modulating induced pluripotent stem cells-cardiac myocytes (iPSC-CM) metabolism by switching substrates and changing metabolic pathways, you can decrease iPSC-CM heterogeneity and arrhythmogenicity. Novel culture methods and tissue engineering along with animal models of heart failure are needed to fully unlock the potential of cardiac myocytes derived from induced pluripotent stem cells for cardiac regeneration.

**Abstract:**

Heart failure (HF) is a common disease in which the heart cannot meet the metabolic demands of the body. It mostly occurs in individuals 65 years or older. Cardiac transplantation is the best option for patients with advanced HF. High numbers of patient-specific cardiac myocytes (CMs) can be generated from induced pluripotent stem cells (iPSCs) and can possibly be used to treat HF. While some studies found iPSC-CMS can couple efficiently to the damaged heart and restore cardiac contractility, almost all found iPSC-CM transplantation is arrhythmogenic, thus hampering the use of iPSC-CMs for cardiac regeneration. Studies show that iPSC-CM cultures are highly heterogeneous containing atrial-, ventricular- and nodal-like CMs. Furthermore, they have an immature phenotype, resembling more fetal than adult CMs. There is an urgent need to overcome these issues. To this end, a novel and interesting avenue to increase CM maturation consists of modulating their metabolism. Combined with careful engineering and animal models of HF, iPSC-CMs can be assessed for their potential for cardiac regeneration and a cure for HF.

## 1. Introduction

Heart failure (HF) is a common disease in the Western world with a high prevalence and steadily rising incidence [1]. HF occurs when the heart cannot meet the metabolic demands of the body. HF can be caused by congenital defects or be acquired later in life and is often a lethal disease, with the only cure being cardiac transplantation [1]. Because there is a critical shortage of donor hearts, most therapies involve slowing the progression of the disease by managing the symptoms [1]. Stem cell therapy is a promising new approach to generate cardiac myocytes (CMs) to repair failing hearts [2]. To date, a wide variety of different stem cell types have been investigated for their ability to repair the failing heart in both animal studies and human clinical trials [3,4,5,6,7,8,9,10,11]. The emergence of induced pluripotent stem cell (iPSC) technologies is an additional cell source with therapeutic potential to treat HF. The major advantage of iPSC over other cell types for cell transplantation is that not only are the cells are patient-specific, thereby circumventing the important issue of tissue rejection associated with transplantation procedures, but they can also be differentiated into cardiac tissue [12]. Yet, despite the encouraging results from preclinical studies, a number of obstacles still need to be overcome before iPSC-based technology can be applied to animals for therapeutic purposes [13].

Principal limitations related to the use of iPSC-derived cardiac myocytes (iPSC-CMs) for regeneration include the heterogeneity of the cardiac cell types generated by the differentiation protocols and their lack of maturity [12]. Currently, all known differentiation protocols lead to a heterogeneous population of cells with varying proportions of atrial-, ventricular-, and nodal-like myocytes, as well as non-myocytes which, if implanted, are an arrhythmogenic risk [14,15]. Furthermore, because differentiation protocols can have undifferentiated pluripotent stem cells in the cellular milieu, there is concern that implantation of undifferentiated cells can contribute to formation of teratomas [16,17]. Therefore, efficient purification and enrichment strategies for CMs as well as cardiac subtypes of interest are necessary [18].

The lack of maturity of the CMs generated by current differentiation protocols is also an important limitation [12,19]. Characterization of their structure, gene expression and electrophysiological properties indicate that they are more like embryonic or fetal than adult CMs. Ongoing studies are now optimizing techniques allowing for maturation of the cells. Most of them attempt to recapitulate a realistic reconstruction of the in vivo myocardium. Cardiac tissue engineering aims at reconstituting mature cardiac tissue with thick myocardial structures in vitro whose structure can be maintained in vivo. Therefore, researchers have been focusing on the role of the extracellular matrix (ECM). Major changes in ECM occur during normal development, thus influencing in vitro differentiation [20]. Furthermore, because the heart is a mix of myocytes and non-myocytes, researchers have been culturing CMs with non-myocytes including endothelial cells, smooth muscle cells, and fibroblasts to recapitulate the native environment. Because the heart is constantly in motion, iPSC-CMs have also been subjected to mechanical stretch in an effort to elicit an adult phenotype [21,22].

More recently, researchers have focused their attention on iPSC-CM metabolism. It is now known that defects in cardiac metabolic pathways could have pathological consequences like hypertrophy and failure [23,24,25,26,27,28]. Moreover, cardiac myocyte metabolism changes drastically as HF progresses [23,27,29,30,31]. In hypertrophied and failing hearts, it is well established that expression patterns related to the metabolic pathway component show a switch from adult- to fetal-like profile [32,33]. Mitochondrial oxidative metabolism decreases while anaerobic glycolysis and ketone body utilization increases. Consequently this metabolic shift results in reactive oxygen species (ROS) liberation, accumulation of lactate and lipids, and decreased adenosine triphosphate (ATP) levels [27,31,34,35].

In adult CMs, the dominant metabolic pathway is fatty acid oxidation, whereas in fetal CMs glycolysis predominates [36]. Recently, studies done on iPSC-CMs have demonstrated that these cells primarily rely on glycolysis for energy production, thus resembling more fetal CMs than adult CMs. To be used as a relevant in vitro model to study disease processes as well as a therapeutic for HF, there is an urgent need to mature iPSC-CM metabolism toward a more adult-like adult cardiac myocyte phenotype [36,37,38,39,40].

Regardless of their maturity, researchers are currently studying the potential of iPSC-CMs for cardiac regeneration. Animal models such as rodents, canines, pigs, and primates are used [11,16,41,42,43,44,45,46,47,48,49,50,51]. So far, some studies have shown encouraging results after iPSC-CM engraftment like long-term cell survival, increased cell maturation and contractile improvement while others have found the opposite. Additionally, almost all have found the development of post-transplant cardiac arrhythmias [46,49,50,51].

The aim of this review, therefore, consists of (a) gathering the last strategies employed to purify or enrich cell populations in CMs or/and chamber-specific CMs from iPSCs, (b) the techniques used to improve their maturation, (c) the different techniques to modulate iPSC-CMs metabolism, (d) discuss the importance of HF animal models and iPSC-CMs engineering for cardiac regeneration studies.

## 2. Cardiac Differentiation Protocols

The initial differentiation protocols involved the generation of embryoid bodies, a spherical collection of cells containing all three germ layers: ectoderm, endoderm, and mesoderm [52,53,54]. Follow up studies soon discovered iPSC-CMs could be generated from monolayers of iPS cells [15,55]. Regardless of whether embryoid bodies or monolayers are used, differentiation protocols seek to recapitulate the signaling cascades that occur during normal development.

Cardiac progenitor cells are thought to originate from the mesoderm after differentiation is induced via signaling cues from adjacent tissues [56]. Activation of tumor growth factor-β (TGF-β) signaling cascades promote cardiac mesoderm formation initially, while inhibition of the Wnt/β-catenin pathway is necessary for further cardiac differentiation. Consequently, a variety of protocols seek to replicate this process in vitro with the goal of generating ventricular myocytes. Some protocols modulate the Wnt/β-catenin signaling axis using members of the TGF-β super family, some protocols use small molecules, and some use a combination of the two along with other cytokines such as basic fibroblast growth factor (bFGF), vascular endothelial growth factor (VEGF) and dickkopf homolog 1 (DKK1) [15,55,57,58,59,60,61,62,63,64,65,66,67]. Because insulin is known to inhibit cardiac differentiation, but enhances cardiac proliferation, differentiation is often started in insulin-free media followed by insulin-containing media after cardiac lineage commitment [68,69,70,71,72,73].

Interestingly, it was found that vitamins could influence the efficiency and specificity of the cardiac progenitor and differentiated cells. For instance, the addition of ascorbic acid (vitamin C) in combination with other factors such as human serum albumin and RPMI (Roswell Park Memorial Institute) medium during the cardiac differentiation protocol has been shown to promote cardiac differentiation of pluripotent stem cells [68,74,75,76]. Also, retinoic acid, the biologically active form of vitamin A, is known to play a role in the control of atrial-specific gene expression and its exclusion is important for specification of the ventricles [77]. Consequently, Zhang et al. showed that retinoic acid regulates fate specification of mouse embryonic stem cells (mESCs) towards an atrial phenotype [78]. It is also possible to generate Purkinje and pace-making cells adding specific cytokines to the media at certain time points.

For example, in 2002 Rentshler and coworkers showed that neuroregulin-1 (NRG-1), an important regulator of both cardiac development and postnatal function, could convert murine embryonic contractile myocytes into conducting cells [79]. Later, other studies have demonstrated that NRG-1 and the receptor tyrosine kinases ErbB2, ErbB3 and ErbB constitutes a critical regulator in the development of specialized nodal and conduction structures [80,81]. Moreover, in 2010, Laflamme and colleagues showed that NRG-1/ERBB signaling regulates the ratio of nodal- to working-type cells and the inhibition of this pathway orientates cells towards a nodal phenotype in differentiating human ESC-derived CM (hESC-CMs) cultures [82].

Additionally, endothelin, a paracrine factor abundant in the arterial system of the heart and secreted by endothelial cells, appears to be involved in Purkinje/nodal cell differentiation. At the embryonic stage, all the myocytes express receptors for endothelin. Bond et al. showed that ET-1 treatment of embryonic avian CMs induced an increased expression of Wnt11 and Wnt7a, suggesting a role of the Wnt proteins in the differentiation of the conduction system [83]. Gassanov et al. further demonstrated that ET-1 increased the percentage of pacemaker-like cells of ESCs into cardiomyocytes during cardiac differentiation protocols [84]. In addition, a co-culture of hiPSCs in a serum-free medium with the visceral endoderm-like cell line END-2, which secretes endothelin, resulted in the generation of not only nodal-like cells but also atrial-like cells [85].

Unfortunately, the limitations of all differentiation protocols are inconsistencies and low reproducibility [64,86,87,88]. This can occur not only between different cell lines, but also the same cell line at different passage numbers. While evidence suggests that the phase of the iPS cell cycle at the time of directed differentiation probably influences the yield of CMs, another strategy to overcome this limitation is purifying myocytes from non-myocytes [64].

## 3. Purifying Induced Pluripotent Stem Cells-Cardiac Myocytes (iPSC-CMs) for Cardiac Regeneration

Cardiac differentiation protocols have improved significantly over the last two decades. However, obtaining pure cultures of IPSC-CMs is still an issue. This is a barrier for their therapeutic use because undifferentiated cells may produce tumors. Furthermore, non-conducting cells can pose an arrhythmogenic risk [89,90,91,92]. Depending on how the non-conducting cells alter the geometrical arrangement of conducting cells, an anatomical or functional reentrant circuit may form [93]. Consequently, a variety of strategies exist to purify iPSC-CM cell cultures.

Some of the first protocols used to purify iPSC-CM cultures involved manually dissecting beating areas from non-beating areas [54,94]. Another early method consisted in using a low viscosity density gradient, Percoll, with low centrifugal forces. The problem with both these methodologies are scalability and purity, with up to 30% of the cells in the culture being non-myocyte [95].

In 2010, a non-genetic strategy for CM purification was reported by Fukuda and colleagues as an alternative strategy [96]. The specific and non-toxic tetramethylrhodamine methyl ester perchlorate (TMRM) incorporates reversibly into the mitochondria which are very abundant in CMs. Thus, using TMRM staining and sorting based on mitochondrial fluorescence allows for separation of CMs with very high mitochondria content from the non-myocyte cells with low mitochondria number.

A unique and more recent strategy for purifying iPSC-CMs is lactate selection. While almost all mammalian cells use glucose as their energy source, the brain, heart, and muscle can use lactate. Consequently, Tohyama et al. discovered that switching iPSC-CMs from a glucose- to a lactate-containing media preferentially allowed for the survival of iPSC-CMs and the death of non-CMs [97].

Other purification protocols use transgenes. Transgenes under the control of cardiac-specific promoters can be used to purify iPSC-CMs by antibiotic selection or fluorescence-activated cell sorting (FACS). Transgenes are most delivered to iPSC-CM cell cultures by lentiviral or adenoviral vectors. Examples of commonly used promoters are the fast myosin heavy chain (α-MHC; encoded by *MYH6*) or the slow myosin heavy chain (β-MHC; encoded by *MYH7*) promotors [98,99,100]. Additionally, chamber-specific promoters can be used as well. Both Huber et al. and Bizy et al. used this approach to purify ventricular- and atrial-like iPSC-CMs derived from either EB formation or monolayers using respectively the myosin light chain-2v promotor and the myosin light chain-2a promotor [100,101]. While viral reporters are robust, efficient, and inexpensive to use in the laboratory, the use of viral vectors poses a hindrance to clinical translation.

A unique approach to identify and purify atrial-like cells was developed by Josowitz et al. [102]. They performed electroporation of hiPSCs with a bacterial artificial chromosome (BAC) reporter construct in which the fluorescence was driven by expression of the atrial-specific gene sarcolipin (SLN), a small Ca^2+^-binding protein predominantly found in the atria, and they were able to successfully isolate hiPSC-derived atrial-like cardiomyocytes.

A less-invasive approach than viral vectors is the use of a mRNA-based approach called molecular beacons. Molecular beacons are synthetic oligonucleotides whose structure contains a quenched fluorophore whose fluorescence is restored when they bind their target DNA/RNA sequence. Used with FACS, this approach has been used to purify subpopulations of myocytes. Nevertheless, as with the use of transgenes, it involves the transfer of foreign nucleotides into the myocyte [103].

Another less-invasive approach to purify iPSC-CMs is the use of magnetic beads. The commercialized MACS Miltenyi Biotec^®^ kit is used as a two-step protocol based on magnetic separation [104]. Nanometer-size magnetic beads coated with specific antibodies can be used to separate a cell type of interest. Multiple groups have used this protocol to achieve cultures with greater than 90% iPSC-CM purity [101,105,106,107]. Interestingly, using a high-throughput flow cytometry screen in Nkx2-5–GFP differentiated cultures, Dubois et al. were able to identify a cell-surface receptor, the signal-regulatory protein alpha (SIRPA) that is only expressed on hPSC-CMs as well as on human fetal CMs [105]. Using magnetic bead sorting for selection of SIRPA-positive cells, they were able to isolate populations consisting of up to 98% CMs, identical to what they found using FACS.

While other cell surface markers such as the hyperpolarization-activated cyclic nucleotide-gated channel 4 (HCN4), which encodes for the pace-making current, or platelet-derived growth factor receptor A, a tyrosine kinase expressed on many cell surfaces, have been used in the past to identify iPSC-CM progenitor cells, cluster differentiation 82 (CD82), a tetraspanin family glycoprotein, transiently expressed in late-stage mesoderm cells during iPSC-CM differentiation appears to be another relevant cell surface marker [108]. However, CD77(+)/CD200(−) cell surface signature appears to be efficacious for stem cell derived cardiac myocytes in certain cell lines [109]. Nevertheless, because it is used to identify progenitor populations and not iPSC-CMs, the purity of CMs will depend on the differentiation protocol and additional rounds of selection. Thus, antibody-based cell enrichment to surface marker proteins is one potential avenue to purify iPSC-CMs using cell sorting without introducing foreign nucleotides.

## 4. Maturing iPSC-CMs

While there is no clear consensus on which differentiation protocol or which purification protocol to use, there is also no clear consensus on what culture day to utilize the cells. This is somewhat problematic because the iPSC-CMs phenotype changes overtime, with older iPSC-CMs displaying a more mature phenotype [110,111,112,113,114,115,116].

Regardless, compared to adult myocardium, iPSC-CMs have notable differences with adult CMs in their structure and morphology, electrophysiology, excitation–contraction coupling, as well as in their metabolism. Table 1 recapitulates the main characteristics and markers associated with immature vs. mature CMs. Immature CMs refers to human fetal ventricular CMs and iPSC-CMs. Mature CMs refers to human adult ventricular CMs.

### 4.1. Structural and Morphological Properties

Structurally, adult myocytes are anisotropic, with a rod-like morphology. They have well-defined sarcomeres, with myosin molecules overlapping actin filaments at regular intervals between the Z-lines. Also contained in the sarcomere is the troponin/tropomyosin complex, myosin-binding protein c, and titin, which is the largest protein in the body acting as a biological spring [117]. T-tubules invaginate the membrane at the Z-disk near the sarcoplasmic reticulum (SR). Within the sarcomeres are a network of mitochondria [93].

Compared to adult myocytes, iPSC-CMs have disorganized myofibrils associated with decreased levels of the intermediate filament protein desmin, which regulates sarcomeric architecture as well as the position of the nucleus [54,118,119]. While iPSC-CMs can be bi- or multi-nucleated like adult cardiac myocytes, the majority are mono-nucleated [120,121]. iPSC-CMs also express a variety of fetal sarcomeric proteins. For example, instead of expressing the adult form of the myosin, β-MHC, they express the fetal isoform, α-MHC [115,122,123,124]. As fetal CMs, iPSC-CMs express to a high degree the myosin light chain 2a (MLC-2a) and display low expression of the myosin light chain 2v (MLC-2v) [125,126]. IPSC-CMs also express the fetal titin isoform and the skeletal form of regulatory troponin I (TnI) instead of the cardiac isoforms [127,128].

### 4.2. Electrophysiological Characteristics

Compared to the adult cardiomyocyte which have a resting membrane potential of approximately −90 mV, iPSC-CMs can have a maximum diastolic potential of approximately −60 mV early after differentiation initiation but can become more negative with culture time [129,130]. iPSC-CMs also express a high level of the hyperpolarization-activated cyclic nucleotide-gated channel 4, the pacemaker current, *I* funny (*I*_f_), leading to a high degree of automaticity iPSC-CMs likewise have been reported to express lower levels of KCNJ2, the main subunit of the inward-rectifier potassium current (*I*_K1_). However, these findings are disputed with some studies finding no difference in inward-rectifier potassium current [131,132]. iPSC-CMs also express the fetal form of SCN5A, which encodes the α-subunit of the cardiac sodium channel [133]. This results in a slower action potential upstroke velocity. The reduced upstroke velocity and the circumferentially disrupted gap junctions (connexin 43; encoded by *GJA1*) result in a slower conduction velocity in the engineered tissue when compared to a healthy adult myocardium [101,134].

During the plateau phase of the action potential, calcium enters the myocyte through the L-type calcium channel (Cav1.2, *CACNA1C*). Compared to adult myocytes, iPSC-CMs have been reported to have lower levels of the L-type Ca^2+^ channel (LTCC) β-subunit (Cavβ2, *CACNB2*), the SR calcium release channel, the ryanodine receptor 2 (RYR2), as well as an absence of the SR calcium sequestering protein calsequestrin 2 (CASQ2) [131,135].

### 4.3. Metabolic Properties

Induced pluripotent stem cells primarily rely on glycolysis to meet their metabolic demands [37,38]. Evidence shows that the success of somatic cell reprograming requires a metabolic switch, with a reduction in oxidative phosphorylation and an increase in glycolysis. iPS cells also need glutamine, threonine, and methionine to maintain their pluripotent state in cell cultures [136,137,138].

Glutamine is converted into glutamate, which can be utilized in the production of the antioxidant, glutathione, as well as enter the tricarboxylic acid (TCA) cycle after being converted into α-ketoglutarate [136]. Likewise, threonine is broken down into pyruvate and α-ketobutyrate, which also enters the TCA cycle [138]. While, methionine can enter the TCA cycle, it is believed to control iPSC maintenance and pluripotency via its metabolite, S-adenosylmethionine, a universal methyl donor [137,139]. Consequently, methionine is a major epigenetic regulator of iPSC pluripotency. Interestingly, both glutamine and threonine can regulate S-adenosylmethionine levels through their metabolites α-ketoglutarate and glycine [140,141,142].

**Figure 1:**
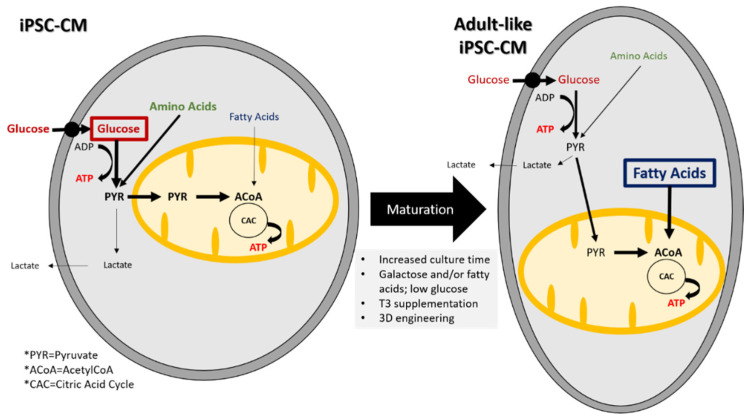
Substrates and metabolic pathways in iPSC-CMs and adult-like iPSC-CM. Metabolic changes are required for maturation into adult-like iPSC-CMs.

Characterization of iPSC-CM metabolism shows that they mostly rely on glucose and amino acids and to a lesser extent on fatty acids whereas in adult CMs, the dominant metabolic pathway is fatty acid oxidation [143,144,145,146]. Figure 1 compares the different metabolic substrates and pathways used by iPSC-CMs and adult CMs.

Remarkably, despite their immature phenotype, iPSC-CMs recapitulate phenotypes found in patients with monogenetic and polygenetic heart diseases, making them an excellent platform for disease modeling [107,116,128,133,147,148,149]. For example, even though iPSC-CMs have an increase in the funny current, Benozoni et al. found that iPSC-CMs derived from 3 siblings with persistent untreatable atrial fibrillation AF had an increase in the funny current along with changes in the calcium currents, suggesting a plausible molecular mechanism [147].

### 4.4. iPSC-CM Maturation

Many strategies have been developed and are currently employed so that iPSC-CMs can not only be similar to adult CMs but also be employed safely for clinical applications. One of the first and simplest strategies is to let the myocytes age. Studies have shown that in the first month of culture, iPSC-CMs increase in cell size and their proliferation reduces [54,118,150,151]. In addition, they showed an increased sarcomere organization, a reduction in the maximum diastolic membrane potential, larger calcium transients, and an increase in contractility [131,146]. Another methodology to maturing iPSC-CMs is to use biophysical cues. For example, adult cardiac tissue has an elastic modulus of approximately 10 kPA. Standard tissue culture plastic in contrast has an elastic modulus greater than 100 kPA [124]. By culturing iPSC-CMs in hydrogels with an elastic modulus similar to physiological conditions, a more mature phenotype is obtained. Likewise, because adult myocytes are rod-shaped, it has been demonstrated that plating iPSC-CMs on micropatterns that force them to take on an adult shape resulted in a more negative resting membrane potential, increased upstroke velocity, longitudinal calcium propagation, increased myofibril alignment, and increased contractility [152,153]. Additionally, it has been shown that by mechanically stressing iPSC-CMs or electrically stimulating them can shift the phenotype into a more adult-like phenotype [154,155,156].

It is also becoming clear that culturing iPSC-CMs with additional cell types is necessary to elicit an adult phenotype. Some of the first co-culturing techniques involved co-culturing iPSC-CMs with fibroblasts [94,157]. However, the optimal fibroblast cell type needed to elicit an adult-like phenotype are cardiac fibroblasts formed via the epithelial-mesenchymal transition (EMT) and originating from epicardial cells. Constructs made with co-cultures of iPSC-CMs and EMT fibroblasts had better systolic and diastolic benchmarks when compared to tissues made from iPSC-CMs and other stromal cells [158]. Likewise, adding endothelial cells and/or smooth muscle cells with the fibroblasts also improves the tissues’ phenotype [159,160].

Recently, Giacomelli et al. showed that tri culture of iPSC-CMs, cardiac fibroblasts, and cardiac endothelial cells can result in maturation of excitation-contraction machinery resulting in enhanced contraction in a scaffold free 3-D microtissues [161]. They also found tri-culture profoundly altered iPSC-CM metabolism compared to co-culture.

## 5. iPSC-CM Metabolism

Myocytes, like all mammalian cells, primarily obtain their energy from the oxidation of fats, sugars, and amino acids via the citric acid cycle [145]. Carbon sources are broken down into acetyl-CoA which is converted into citrate. Then through a series of redox reactions, the reduced nicotinamide adenine dinucleotide (NADH) and the reduced flavin adenine dinucleotide (FADH_2_) are generated. NADH and FADH_2_ then enter the electron transport chain, where they are involved in the synthesis of ATP by oxidative phosphorylation. Because the yield of ATP is dependent on the number of carbon atoms that are broken down into acetyl-CoA, oxidation of 1 g of triacylglycerol can produce up to 6 times the amount of ATP as does the oxidation of 1 g of hydrated glycogen [162].

To assess iPSC-CM metabolic maturity, it is important to compare them to both fetal and adult CMs. In utero, the fetal heart is adapted to a hypoxic environment which is low in fatty acid content. Consequently, the fetal heart relies primarily on glucose and lactate to meet its energy demands [163,164,165,166]. Shortly after birth, the fetal heart undergoes a metabolic switch to fatty acid oxidation. Associated with this are increases in the expression of peroxisome proliferator activated receptor α (PPARα) and PPAR coactivator 1α (PGC-1), both of which have been shown to be involved with mitochondrial biogenesis [167,168]. An increase in their expression leads to increased mitochondrial biogenesis and in an increase in carnitine palmitoyl transferase I (CPT I), an enzyme critical for fatty acid metabolism [168,169,170]. While endurance exercise can increase the amount of PPARα, a variety of pathological conditions can inhibit it. For example, both chronic hypoxia and ischemia caused by coronary artery disease can reduce its expression [34,37,171]. In fact, in a variety of heart diseases there is metabolic maladaptation and, consequently a return to fetal metabolism [23,29,30,35].

Because it has limited storage capacity, the adult heart has evolved to be a metabolic omnivore, capable of utilizing mainly fatty acids, but also glucose, lactate, branch chain amino acids, and ketone bodies to meet its energy demands [145]. To obtain the oxygen for the oxidation of the various carbon sources, the adult heart relies on alterations in coronary blood flow. There is a constant crosstalk between the myocardium and coronary arteries, where even simply increasing the coronary blood flow outside of a change in heart rate is enough to increase myocardial oxygen consumption [168,172].

Induced pluripotent stem cells undergo a metabolic switch during differentiation into CMs. For example, Rana et al. found that ATP levels in iPSC-CMs increased over 21 days in culture [173]. Furthermore, they found that the carbon source supplied in the media exerted the largest influence on the amount of ATP. Media containing galactose or galactose and fatty acids resulted in the biggest increase in ATP. The galactose and galactose with fatty acid medias also resulted in a decrease in oxygen consumption, indicating an increase in oxidative phosphorylation. Different strategies can be used to increase iPSC-CM maturation by modulating their metabolism. These are recapitulated in the Figure 1. A follow-up study by Correia et al. discovered that by culturing iPSC-CMs in media containing galactose, oleic acid, and palmitic acid, they could improve iPSC-CM maturity [38].

By switching the substrate from glucose to another carbon source, Correia et al. found they were able to induce a faster iPSC-CM action potential upstroke, an increase in the peak calcium amplitude, faster calcium reuptake times, an increase in peak contraction and faster relaxation kinetics. Interestingly, without a sugar source, fatty acids alone induced lipotoxicity. Nevertheless, media containing fatty acids with a sugar substrate prevented the accumulation of lipid intermediates and lipotoxicity. The combination of fatty acids with a non-glucose sugar substrate ultimately resulted in improved cellular oxidative capacity as was a transcriptional signature closer to adult human ventricular myocytes.

Another study found that the increase in oxidative phosphorylation caused by glucose deprivation and fatty acid supplementation was associated with the inhibition of hypoxia inducible factor 1α (HIF1α) and its downstream target, lactate dehydrogenase A (LDHA) [37]. Importantly, when they silenced HIF1α or LDHA with either small molecules or small interferent RNA (siRNA), the iPSC-CMs underwent a metabolic switch from aerobic glycolysis to oxidative phosphorylation. Accompanying this shift was an increase in the amount of PPARα, β-oxidation, and a shift in iPSC-CM phenotype. As with the glucose deprivation experiments, inhibition of HIF1α or LDHA resulted in an increase in the peak calcium transient and peak force development. Additionally, they found a lower resting membrane potential, another marker of maturation.

An alternative way to alter iPSC-CM metabolism is to add the thyroid hormone, triiodothyronine (T3), to the cell cultures [169,174]. T3 is synthesized from the prohormone, thyroxine (T4). In rodents, T3 levels remain low until after birth and in humans T3 steadily increases after 30 weeks of gestation [170,175]. T3 binds its nuclear receptor (TR). The TR includes a zinc finger motif DNA binding domain and a COOH-terminal domain that can mediate interactions with other ligands, transcription activators, and transcription repressors. In addition to regulating the metabolism of fatty acids, it also upregulates the expression of the MHCα, the Na^+^/K^+^-ATPase, the Na^+^/Ca^2+^ exchanger, voltage-gated potassium channels (Kv1.5, Kv4.2, Kv4.3) and the sarcoplasmic reticulum Ca^2+^-ATPase (SERCA2a) while downregulating the expression MHCβ and SERCA2-A regulator phospholamban (PLB). When applied to iPSC-CMs, the results comprised increased T-tubule development, faster calcium transient kinetics, faster AP kinetics, and an increase in maximum tension [169,176].

Interestingly, changes in metabolic enzymes are chamber-specific. While atrial pressures remain the same during gestation, both left and right ventricular pressures steadily increase. However, left vs. right pressure gradients do not form until after birth [177,178]. Because the ventricles contract against larger pressures, and hence do more work, they have different expression of metabolic enzymes than the atria [179]. Compared to the ventricle, the atria expressed lower levels of hydroxyacyl-CoA-dehydrogenase, citrate synthase, malate dehydrogenase and lactate dehydrogenase [180]. Additionally, amino acid metabolism was found to be different between the atria and ventricles, with the ventricle utilizing lower levels of aspartate, glycine, and proline [181].

Interestingly, simply culturing iPSC-CMs in 3D engineered tissue constructs has been found to increase mitochondrial biogenesis and decrease in anaerobic glycolysis [182,183,184]. Anaerobic glycolysis is the process where glucose is transformed into lactate when limited amounts of oxygen are available. However, one important caveat is that the constructs had to be “exercised” either by electrical field stimulation or by having to contract against a mechanical load.

While great care has been taken to make iPSC-CMs as similar to adult CMs as possible, their true promise is whether or not they can be used as a therapeutic for the treatment of cardiovascular disease.

## 6. Transplantation of iPSC-CMs for Treatment of Heart Failure

Heart failure often presents itself at an older age and has a five-year mortality rate of 42.3% [1]. Despite decades of basic science research, the development of novel pharmaceuticals, and improvements in whole organ transplantation, the five-year mortality has remained almost constant in the 21st century. Therefore, iPSC-CMs have the potential to become a 21st-century medical technology that can save many lives.

Despite the fact HF can occur in any mammalian species, it is also uncommon in a lot of species. More frequent occurrences are in canines, felines, primates, and humans [185,186,187,188]. Despite several limitations regarding the use of small animals, rodents are commonly used to mimic the pathophysiological aspects of HF. Small animal models are generally generated by genetic modification, by surgery, or by administration of drugs which have toxic effects [189]. Large animals including porcine, ovine, canine, feline and primates are more costly, require larger facilities but offer important advantages over small animals since they are physiologically closer to humans and mimic human HF features [190]. Importantly, the cause of HF differs between species. While the most widespread form of HF in felines is the result of hypertrophy, in small canines the primary cause is valvular heart disease while in large canines it is dilation [185]. Although numerous pharmacological interventions exist to treat the symptoms, none treat the underlying cause. Whereas the right in vitro culture conditions are still being worked out to produce the optimal cellular therapy for the treatment of HF, numerous pioneering studies have already been conducted.

A wide variety of cell-based therapies have been tested to treat ischemic heart disease and HF. For example, bone marrow mononuclear cells, hematopoietic stem cells, endothelial progenitor cells, as well as cardiac progenitor cells, and both embryonic- and induced pluripotent stem cell-derived CMs have been tested as a cellular therapy for HF [8,16,42,43,191,192,193,194,195,196]. In addition, different strategies are adopted for cardiac regeneration using stem cell-derived CMs. Cells can then be injected as single cells, patches, or sheets [11,43,47,155,160,197,198,199]. Almost all improved some metrics of cardiac function, suggesting paracrine factors excreted from grafts exert some regenerative capacity. However, it is debatable if they will have any long-term meaningful clinical effect, especially in non-rodent hearts [200].

For example, in non-human primates, Chong et al. initially found that injection of hESC-CMs extensively remuscularized infarcted host heart tissue [196]. However, increases in the ejection fraction were highly variable with some animals showing improvement and others showing no improvement at all. The hESC-CMs injected groups also had arrhythmogenic complications.

A follow up study by Shiba et al. found that injecting allogenic iPSC-CMs in infarcted primate hearts can increase cardiac contractility [46]. While the treated group had ventricular arrythmias, they were transient, disappearing 12 weeks after injection. They hypothesized that in vivo maturation of their grafts was responsible for the cessation of arrythmias.

Further work by Kashiyama et al. found that by grafting major histocompatibility complex (MHC)-matched allogeneic iPSC-CMs cardiac function can be improved and preserved for up to six months after transplantation [42]. Interestingly, they also found significant improvement in the MHC-mismatched group as well.

Another study by Zhu performed in non-human primates, pluripotent stem cell-derived cardiovascular progenitor cells (hPSC-CVPCs), defined as iPSCs harvested after three days of differentiation, and injected into the myocardial infarction group, resulted in an increase in left ventricular function at the 28-day time point [200]. However, after 140 days, no viable hPSC-CVPCs could be detected probably because of immunorejection. Therefore, in non-human primates it appears that a combination of paracrine factors, remuscularization, and immune responses contribute to a transient increase in cardiac output. However, it has yet to be established if they will have any sustained long-term improvements.

To evaluate the potential of hiPSCs for regeneration purposes, many studies have focused on the study of the immunogenicity of iPSC-derived cells. For example, in 2011, a study from Zhao et al. showed that unlike ESCs, some cells differentiated from iPSCs could induce a T cell-dependent immune response in a subgroup of patients [201]. A different study led by Araki et al. in 2013 demonstrated that transplantation of terminally differentiated cells from ESCs and iPSCs induced low or no immune response and no increase in immunogenicity gene expression thus suggesting a limited immunogenicity of transplanted cells differentiated from both ESCs and iPSCs [202]. Another study performed in humanized mice by Zhao et al. demonstrated that some in vitro differentiated cell types are less immunogenic than others [203]. Interestingly, different levels of hiPSC-derivatives maturity can explain the expression of certain immunogenic antigens and the generation of T cell-dependent immune response. Altogether, these results show that immunogenicity of differentiated cells highly depends on the hiPSC clones selected for differentiation as well as on the cell type that is generated and its maturity level. For a thorough review, see Tapia and Schöler, 2016 [204].

Regardless of the animal model or the clones selected, it is still being debated whether direct injection or the transplantation of iPSC-CM sheets are more beneficial because they all seem to improve cardiac function, at least transiently [42,43,46,47,48,50,193,199,200,205,206]. While direct injection can allow the injection of cells directly into the damaged tissue, because the heart is in constant motion, iPSC-CMs embedded in scaffolds should allow for better retention. However, because of the motion, developing iPSC-CM sheets that mimic the biomechanical properties of the heart both during systole and diastole has been challenging [207,208].

Recently, a proof-of-concept study has been demonstrated in mouse hearts. Cui et al. used beam-scanning stereolithography to print a four-dimensional (4D) cardiac patch with physiological adaptability [197]. They called it a 4D patch because after forcing light-induced graded internal stress, they could use a solvent to induce relaxation of the material and autonomous morphing of the scaffold to recapitulate with high fidelity the curvature of the heart. As a result, the patch’s fiber mesh was able to change in accordance with the diastole and systole of the cardiac cycle. By reproducing the anisotropy of healthy cardiac tissue, they hypothesized their patches would more effectively exchanges of nutrients and metabolites, as well as guided contraction for the engineered cells. After seeding the patches with a variety of iPSC-CMs, hECs, and human multipotent stem Cells (hMSCs) and implanting the tissue into infarcted hearts, they found increased cellular engraftment, revascularization of the infarcted tissue, and maturation of the iPSC-CMs. However, they did not find evidence the patch functionally integrated and increased the ejection fraction via force generated by the patch. Therefore, the effects are most likely due to the excretion of paracrine factors. Nevertheless, it is unknown if the patches will functionally engraft in larger animal hearts.

Given the promising results of the basic science research and the limited treatment options for HF, human trials are commencing. The first human trial of human ESC-derived cardiac progenitors was conducted in 2014 (NCT02057900) [11,209]. Because only 10 patients were enrolled in the study and the cells were administered at the time of coronary artery bypass grafting and/or mitral valve repair, which by themselves improves ventricular function, drawing any conclusion of the effectiveness of hESC-CM transplantation to treat ischemic heart disease from this trial is near impossible. Nevertheless, the Transplantation of Human Embryonic Stem Cell-derived Progenitors in Severe Heart Failure (ESCORT) trial demonstrated the technical feasibility of producing clinical grade hESC-derived progenitor cells and their short- and medium-term safety. Consequently, two new clinical trials are underway.

The Treating Heart Failure With hPSC-CMs (HEAL-CHF) (NCT03763136) trial is actively recruiting patients to study if injecting allogenic hiPSC-CMs from healthy donors into the myocardium at the time of coronary bypass grafting in patients with ischemic heart disease can improve myocardial performance [210]. The Biological Ventricular Assist Tissue (BioVAT-HF) (NCT04396899) trial was recently announced May 21, 2020 [211]. The study will be recruiting patients 18 to 80 years of age with an ejection fraction ≤35% in New York Heart Association Class III or IV HF. They will be testing the hypothesis that hiPSC-CM engineered tissue cultures will result in stainable remuscularization and biological enhancement of cardiac function in failing hearts.

## 7. Summary and Conclusions

In the context of HF where cardiac regeneration is the only treatment, iPSC-CMs hold great promise. Nonetheless, they confront important limitations preventing their therapeutic use.

First, the cardiac differentiation protocols do not yield to pure populations of CMs. They are a mix of atrial-, ventricular- and nodal-like cells [15,55,57,58,59,60,61,62,63,64,65,66,67]. Tremendous progress has been made in generating highly enriched populations of hiPSC-CMs through the use of specific cell surface markers for CMs like SIRPA or specific markers for CM progenitors like CD82 [105,108]. While the CD77^+^/CD200^−^ cell surface signature is highly efficient for enrichment of ventricular hESC-CMs, this strategy is not efficient for all hiPSC lines. Thus, generation of enriched chamber-specific hiPSC-CMs is still challenging but necessary since cardiac engraftment of heterogeneous populations could be arrhythmogenic.

Many cardiac purification protocols involve the use of vectors that incorporate their genetic material to the host cells, thus rendering this strategy non suitable for clinical applications [98,99,100,101]. Nevertheless, strategies exist to purify myocytes without altering their genome, with lactate selection gaining popularity [37,40,42,46,97,195,212,213,214,215]. However, they are immature CMs.

Characterization shows they resemble more fetal than adult CMs. Morphologically, iPSC-CMs tend to be less rod-shaped, but rather more rounded or fusiform, whereas adult CMs are typically rod-shaped and elongated. Structurally, their expression pattern of the myosin (light and heavy) is more typical of fetal CMs [115,122,123,124]. Sarcomeres are disorganized and T-tubules are almost nonexistent. Further, iPSC-CMs spontaneously contract and electrophysiological characterization shows a less negative maximum diastolic potential than adult CMs. This results in an action potential amplitude remarkably like fetal CMs (supported by different ionic currents) [101,131,132,133,135].

Studies investigating iPSC-CM metabolism have also found iPSC-CM have a fetal phenotype. iPSC-CMs utilize glucose to meet its energy demands whereas adult CMs preferentially use fatty acids oxidation [37,143,144,145,146,216].

Consequently, several strategies have been employed to mature iPSC-CMs toward a more adult phenotype. Increased time in culture, physical and mechanical stimulation, engineered 3D or 4D constructs, hormonal stimulation and changing carbon substrates in culture media to modulate metabolism have all been shown to shift iPSC-CMs from a fetal to a more adult phenotype [37,40,45,70,101,113,154,155,156,174,176,183,184,216,217,218,219,220,221,222,223].

Influencing metabolism in differentiating CMs from iPSCs might be an inexpensive and valuable solution to improve cell maturity [37,39,40,143,174,216,224]. Furthermore, atria and ventricles exhibit different metabolic enzyme expressions and different amino acid metabolism, with the ventricle utilizing lower levels of aspartate, glycine, and proline [180,181]. Consequently, varying amino acid concentrations in the media might also be a way to selectively metabolically purify atrial iPSC-CMs.

Finally, small, and large animal models of HF are important to assess if iPSC-CMs can be a viable treatment for HF. iPSC-CMs can be transplanted as single cells or sheets [42,43,46,47,48,50,199,200,215,225,226]. Almost all studies, regardless of the animal model used have shown that iPSC-CMs engraft to the host tissue and improve cardiac function, at least transiently. However, their long-term efficacy is unknown. Immunogenicity of the differentiated cells is also another factor that needs to be considered for successful transplantation. As the field continues to evolve rapidly, the complex mechanisms will eventually be understood.

In conclusion, since Yamanaka’s groundbreaking discovery in 2006, iPSCs have been differentiated into CMs and tested as a novel therapeutic for not only the treatment of HF, but a possible cure. While there are still many limitations to their utilization, recent discoveries and technological advances are rapidly accelerating their advancement into the clinic.

## Figures and Tables

**Table 1:** Summary of the principal differences between fetal, adult and induced pluripotent stem cells-cardiac myocytes (iPSC-CMs). iPSC-CMs are more similar to fetal than adult CMs. FA = fatty acids.

Characteristics/Marker	Fetal CMs	Adult CMs	iPSC-CMs
**Morphology**			
Shape	Circular/Irregular	Regular	Circular/Irregular
Cell Volume	Small	Large	Small
**Structure**			
Sarcomeres	Disorganized	Organized	Disorganized
MLC-2v/β-MHC	Low	High	Low
MLC-2a/α-MHC	High	Low	High
Troponin I	Fetal isoform	Adult	Fetal isoform
T-tubules	Absent	isoform	Absent
Gap junctions	Circumferential	Present	Circumferential
Multi-nucleation	Uncommon	Polarized Common	Uncommon
**Electrical**			
Spontaneous contracting	+	-	++
Maximum diastolic	~−40	~−90	~−60
potential, mV			
Action potential amplitude, mV	70–90	110–120	70–90
**Ionic currents**			
I_k1_ inward rectifier	-	+	-
I_kr_ hERG channel	Low	High	Low
I_f_ funny current	High	Low	High
**Metabolism**			
Mitochondria number	+++	+	+++
Metabolic pathways	Glycolysis	FA	Glycolysis
Substrates	Glucose, lactate	oxidation	Glucose, lactate, FA
Oxidative phoshorylation	Low	FA High	Low
